# Trends in the Prevalence of Hepatitis C Infection During Pregnancy and Maternal-Infant Outcomes in the US, 1998 to 2018

**DOI:** 10.1001/jamanetworkopen.2023.24770

**Published:** 2023-07-21

**Authors:** Po-Hung Chen, Lauren Johnson, Berkeley N. Limketkai, Emily Jusuf, Jing Sun, Brian Kim, Jennifer C. Price, Tinsay A. Woreta

**Affiliations:** 1 Division of Gastroenterology & Hepatology, Department of Medicine, Johns Hopkins University School of Medicine, Baltimore, Maryland; 2 Department of Medicine, Johns Hopkins University School of Medicine, Baltimore, Maryland; 3 Vatche & Tamar Manoukian Division of Digestive Diseases, Department of Medicine, David Geffen School of Medicine at UCLA, Los Angeles, California; 4 Krieger School of Arts and Sciences, Johns Hopkins University, Baltimore, Maryland; 5 Department of Epidemiology, Johns Hopkins Bloomberg School of Public Health, Baltimore, Maryland; 6 Division of Gastrointestinal and Liver Diseases, Department of Medicine, Keck School of Medicine of the University of Southern California, Los Angeles; 7 Division of Gastroenterology, Department of Medicine, University of California San Francisco School of Medicine, San Francisco

## Abstract

**Importance:**

Injection drug use is the primary risk factor for hepatitis C virus (HCV) infection in adults. More than one-third of newly reported HCV cases occur in women, particularly among persons aged 20 to 39 years. However, nationally representative data on HCV during pregnancy are limited.

**Objective:**

To estimate the temporal trend of HCV-positive pregnancies during the opioid epidemic and identify HCV-associated maternal and perinatal outcomes.

**Design, Setting, and Participants:**

A cross-sectional study was performed with data from the US, from calendar year 1998 through 2018. Data analysis was conducted from November 14, 2021, to May 14, 2023. Participants included women during in-hospital childbirth or spontaneous abortion in the National Inpatient Sample of the Healthcare Cost and Utilization Project.

**Exposure:**

Maternal HCV infection.

**Main Outcomes and Measures:**

The main outcome was the temporal trend, measured as change in the annual prevalence, in the prevalence of HCV positivity among pregnant women since the start of the opioid epidemic in the late 1990s. Secondary outcomes were the associations shown as relative odds between maternal HCV infection and maternal and perinatal adverse events.

**Results:**

During the study period, more than 70 million hospital admissions resulted in childbirth or spontaneous abortion. Among them, 137 259 (0.20%; 95% CI, 0.19%-0.21%) involved mothers with HCV; these individuals were more often White (77.4%; 95% CI, 76.1%-78.6%), low-income (40.0%; 95% CI, 38.6%-41.5%), and likely to have histories of tobacco (41.7%; 95% CI, 40.6%-42.9%), alcohol (1.8%; 95% CI, 1.6%-2.0%), and opioid (28.9%; 95% CI, 27.3%-30.6%) use compared with HCV-negative mothers. The median age of women with HCV was 28.0 (IQR, 24.3-32.2) years, and the median age of HCV-negative women was 27.2 (IQR, 22.7-31.8) years. The prevalence of HCV-positive pregnancies increased 16-fold during the study period, reaching 5.3 (95% CI, 4.9-5.7) cases per 1000 pregnancies in 2018. Age-specific prevalence increases ranged from 3-fold (age, 41-50 years) to 31-fold (age, 21-30 years). Higher odds of cesarean delivery, preterm labor, poor fetal growth, or fetal distress were associated with HCV-positivity during pregnancy. However, no significant differences were observed in gestational diabetes, preeclampsia, eclampsia, or stillbirths.

**Conclusions and Relevance:**

In this cross-sectional study, the prevalence of HCV-positive pregnancies increased markedly, and maternal HCV infection was associated with increased risks for adverse perinatal outcomes. These data may support recent recommendations for universal HCV screening with each pregnancy.

## Key Points

**Question:**

What were the prevalence and maternal and perinatal outcomes of hepatitis C (HCV)-positive pregnancies during the opioid epidemic?

**Findings:**

This cross-sectional study of more than 70 million births or spontaneous abortions showed the prevalence of HCV-positive pregnancies in the US increased 16-fold between 1998 and 2018. Maternal HCV infection was associated with increased odds of preterm labor, poor fetal growth, or fetal distress.

**Meaning:**

The data from this study suggest that universal HCV screening with each pregnancy may be useful, as the Centers for Disease Control and Prevention propose, but research is needed on the subsequent needs for appropriate specialist care for affected mothers and newborns.

## Teaser

This cross-sectional study examines the prevalence of hepatitis C virus in pregnant women from 1998 to 2018, with a focus on changes since the start of the opioid epidemic in the late 1990s.

## Introduction

Hepatitis C virus (HCV) is the most commonly reported chronic bloodborne infection in the US,^1^ and injection drug use is the primary risk factor for disease transmission among adults.^2^ The US saw a tripling of acute HCV cases between 2010 and 2015,^3^ coinciding with increased heroin (ie, second wave) and synthetic opioid (ie, third wave) overdose deaths during the opioid epidemic.^4^ More than one-third of newly reported HCV cases were among women, and the highest incidence consistently occurred among persons aged 20 to 39 years.^5^ From 2014 to 2017, HCV cases among pregnant women in the US exceeded that of hepatitis B and syphilis combined, despite HCV being the only infection among the 3 not to receive universal screening during prenatal care.^6^ Antepartum opioid use disorder has also more than quadrupled since the start of the opioid epidemic in the late 1990s.^7^ Maternal HCV infection poses potential risks for the fetus, including preterm birth and neonatal death.^6^ Mother-to-child transmission of HCV during pregnancy occurs in approximately 6% of children delivered by women with HCV viremia, and the risk doubles in the setting of poorly controlled HIV coinfection.^8^ Consequently, multiple US medical and public health organizations—including the American College of Obstetricians and Gynecologists—have recently recommended universal HCV screening during each pregnancy.^9,10^

Until lately, data on HCV infection during pregnancy have been scarce, with conflicting reports on the role of HCV in pregnancy and perinatal outcomes. Several newer publications have sought to address the literature gap. Collectively, they suggested an increasing US prevalence of HCV infections during pregnancy over the past 2 decades, although most of these studies reported only on a segment of the period.^6,11,12,13,14^ A few studies also reported adverse perinatal events associated with HCV, although they may not have fully accounted for the potential confounding effects of substance use, which is another known risk for poor pregnancy outcomes.^6,14^

Given increasing HCV infections among women of childbearing age,^5^ the risk of vertical HCV transmission,^8^ and potential pregnancy-related adverse events,^6,14^ clinicians and policy makers need a heightened understanding of HCV infection during pregnancies and its implications on outcomes. Our study aimed to construct the temporal trend of HCV-positive pregnancies in the US since the start of the opioid epidemic in the late 1990s and identify adverse maternal and perinatal results associated with HCV infection.

## Methods

Our report follows the Strengthening the Reporting of Observational Studies in Epidemiology (STROBE) reporting guideline. The Johns Hopkins Institutional Review Board reviewed and acknowledged our study design as not human participant research. Consequently, the need for informed consent did not apply.

The National Inpatient Sample (NIS) is the largest publicly available all-payer inpatient care database in the US and a part of the Healthcare Cost and Utilization Project by the Agency for Healthcare Research and Quality.^15^ The database contains discharge data, including patient demographic data, diagnoses, procedures, insurance payers, hospital characteristics, total charges, and lengths of stay. Before 2012, the NIS was a stratified random sample of approximately 20% of nonfederal, nonrehabilitation hospitals. Starting in 2012, it has approximated a 20% stratified sample of all discharges from participating nonfederal hospitals in the US, excluding rehabilitation and long-term acute care hospitals. Nationwide participation in the NIS has increased over time. As of 2017, 47 states and the District of Columbia contribute to the NIS. Their data are weighted to allow the calculation of national estimates.

We used the *International Classification of Diseases, 9th Revision, Clinical Modification* (*ICD-9-CM*), *International Statistical Classification of Diseases and Related Health Problems, 10th Revision, Clinical Modification* (*ICD-10-CM*), and *International Statistical Classification of Diseases and Related Health Problems, 10th Revision, Procedure Coding System* (*ICD-10-PCS*) codes in the first 40 billing positions to capture all hospital admissions from calendar year 1998 through 2018 that culminated either in childbirth (vaginal and cesarean delivery), stillbirth, or spontaneous abortion (defined as the loss of the fetus before 20 weeks of pregnancy) (eTable 1 in Supplement 1). All included women were aged 18 to 50 years during the pregnancy. Within the cohort, we identified hospitalizations with known diagnoses of HCV positivity. As *ICD-9-CM* and *ICD-10-CM* codes do not specify the absence of a diagnosis, the remainder of hospitalizations presumably were in 1 of 3 categories: (1) HCV-negative, (2) HCV-positive but were unscreened or undetected, or (3) known to be HCV-positive but were uncoded at hospital discharge. For simplicity of nomenclature, we refer to this collective group as HCV-negative.

### Outcomes

Our study examined adverse clinical outcomes that broadly focused on maternal and perinatal events. Maternal outcomes were anemia complicating pregnancy, gestational diabetes, hypertension complicating pregnancy (excluding preeclampsia), preeclampsia or eclampsia, and thyroid dysfunction complicating pregnancy. Perinatal events comprised cesarean delivery, stillbirth, spontaneous abortion, preterm labor (ie, delivery after 20 weeks and before 37 weeks of pregnancy), poor fetal growth, fetal distress, and premature rupture of membranes.

### Covariates

Our models were adjusted for potential confounders (ie, covariates simultaneously associated with HCV infection and maternal and/or perinatal outcomes), which we determined after reviewing a published study design on viral hepatitis-related pregnancy outcomes.^16^ Maternal covariates included age by decade, race and ethnicity, substances used (10 categories including tobacco, alcohol, opioids, and cocaine), HIV, hemodialysis, diabetes, thyroid disorders, hypertension, anemia, primary payer, and median household income for the patient’s zip code. The Healthcare Cost and Utilization Project and its partner organizations defined the racial and ethnic categories for the hospital-reported information in our analysis. We collapsed smaller categories into “other” due to their limited sample sizes. Our analysis adjusted for race and ethnicity as potential confounders, given their possible associations with both the exposure (HCV infection) and the outcome (maternal or perinatal events). In addition, we accounted for medical comorbidities via the validated Charlson-Deyo Comorbidity Index,^17^ using adapted *ICD-9-CM* and *ICD-10-CM* coding algorithms.^18^ We summarized medical comorbidities into 3 severity categories per the Charlson-Deyo Comorbidity Index: 0, 1 to 2, or more than 2. Hospital-related covariates were bed size, location and teaching status, and geographic region.

### Statistical Analysis

Data analysis was conducted from November 14, 2021, to May 14, 2023. We stratified, clustered, and weighted our analyses to navigate the sampling design of the NIS. Additionally, we applied the suggested procedures for multiyear analysis to account for the 2012 NIS sampling redesign.^19^ Categorical variables were compared using Rao-Scott χ^2^ tests for complex surveys.^20^ We performed multivariable logistic regression models to evaluate the relative odds of maternal or perinatal events as a function of HCV infection during pregnancy. Our collective content knowledge informed the model variable selection—no statistical selection procedures aided the process. We used variance inflation factors to identify the presence of possible collinearity among independent variables of the multivariable regression models. All statistical tests were 2-sided and unpaired, and analyses were performed using SAS, version 9.4 (SAS Institute LLC) with α = .05 used as the threshold for significance.

## Results

From 1998 to 2018, more than 70 million hospital admissions in the cohort resulted in vaginal delivery, cesarean delivery, stillbirth, or spontaneous abortion. Of these, 137 259 (0.20%; 95% CI, 0.19%-0.21%) involved HCV-positive mothers (eTable 2 in Supplement 1).

Patient characteristics stratified by HCV status are listed in Table 1. Compared with the HCV-negative group, women in the HCV-positive group were slightly older (median, 27.2 [IQR, 22.7-31.8] years vs 28.0 [IQR, 24.3-32.2] years), more often White (77.4%; 95% CI, 76.1%-78.6% vs 53.9%; 95% CI, 52.6%-55.1%), and generally had a lower socioeconomic status (40.0%; 95% CI, 38.6%-41.5% vs 26.9%; 95% CI, 25.8%-27.9% resided in zip codes with the lowest quartile median household incomes and 74.5%; 95% CI, 73.4%-75.6% vs 39.3%; 95% CI, 38.3%-40.3% received Medicaid). Women who were HCV-positive additionally had more severe Charlson-Deyo Comorbidity Index scores (89.5%; 95% CI, 89.0%-90.0% vs 96.2%; 95% CI, 96.1%-96.3% scored 0) and a higher prevalence of anemia (13.2%; 95% CI, 12.7%-13.8% vs 8.8%; 95% CI, 8.5%-9.1%), but the 2 cohorts did not have clinically meaningful differences in the baseline prevalence of diabetes, HIV, and thyroid dysfunction. Overall, HCV-positive women were significantly more likely to have a history of tobacco (41.7%; 95% CI, 40.6%-42.9% vs 4.0%; 95% CI, 3.8%-4.2%), alcohol (1.8%; 95% CI, 1.6%-2.0% vs 0.1%; 95% CI, 0.11%-0.12%), opioid (28.9%; 95% CI, 27.3%-30.6% vs 0.3%; 95% CI, 0.25%-0.29%), and cocaine (6.9%; 95% CI, 6.4%-7.4% vs 0.3%; 95% CI, 0.26%-0.30%) use.

**Table 1.** Characteristics of Pregnant Women and Hospitals in the US from 1998 to 2018^a^^,^^b^

Characteristic	HCV status, No. (%)	
	HCV-negative (n = 69 901 008)	HCV-positive (n = 137 259)
Age, y		
18-20	7 392 264 (10.6)	5825 (4.2)
21-30	37 894 073 (54.2)	78 684 (57.3)
31-40	23 321 958 (33.4)	49 253 (35.9)
41-50	1 292 712 (1.8)	3497 (2.5)
Race and ethnicity		
Black	7 747 775 (13.8)	8559 (7.1)
White	30 239 399 (53.9)	93 414 (77.4)
Other^c^	18 163 428 (32.3)	18 739 (15.5)
Charlson-Deyo Comorbidity Index		
0	67 271 073 (96.2)	122 813 (89.5)
1-2	2 596 628 (3.7)	13 694 (10.0)
≥3	33 307 (0.05)	752 (0.5)
Diabetes	498 078 (0.7)	1497 (1.1)
HIV	16 762 (0.02)	585 (0.4)
Hypertension	454 605 (0.7)	1322 (1.0)
Thyroid dysfunction	1 477 110 (2.1)	3709 (2.7)
Anemia	6 136 463 (8.8)	18 166 (13.2)
Substance use		
Tobacco	2 814 721 (4.0)	57 263 (41.7)
Alcohol	81 829 (0.1)	2437 (1.8)
Opioids	188 609 (0.3)	39 700 (28.9)
Cannabis	419 522 (0.6)	8209 (6.0)
Sedatives/hypnotics	16 624 (0.02)	1269 (0.9)
Cocaine	193 489 (0.3)	9459 (6.9)
Other stimulants	110 977 (0.2)	6312 (4.6)
Hallucinogens	1869 (0.003)	70 (0.05)
Inhalants	80 (0.0001)	15 (0.01)
Other psychoactives	18 572 (0.03)	3378 (2.5)
Primary payer		
Medicare	387 062 (0.6)	3494 (2.6)
Medicaid	27 404 834 (39.3)	102 013 (74.5)
Private insurance	37 742 800 (54.1)	23 580 (17.2)
Other	4 222 637 (6.1)	7879 (5.8)
Median household income for zip code		
Quartile 1/lowest	13 383 777 (26.9)	49 972 (40.0)
Quartile 2	12 519 516 (25.1)	34 835 (27.9)
Quartile 3	12 274 811 (24.7)	24 997 (20.0)
Quartile 4/highest	11 606 748 (23.3)	15 034 (12.0)
Hospital size		
Small	8 587 316 (12.3)	17 811 (13.0)
Medium	19 092 676 (27.4)	35 901 (26.3)
Large	41 974 613 (60.3)	82 849 (60.7)
Hospital location/teaching status		
Rural	8 084 866 (11.6)	19 154 (14.0)
Urban nonteaching	26 899 867 (38.6)	33 952 (24.9)
Urban teaching	34 669 873 (49.8)	83 454 (61.1)
Hospital region		
Northeast	11 928 810 (17.1)	31 439 (22.9)
Midwest	15 196 028 (21.7)	24 276 (17.7)
South	26 055 748 (37.3)	57 158 (41.6)
West	16 720 422 (23.9)	24 385 (17.8)

Abbreviation: HCV, hepatitis C virus.

^a^ Deliveries and spontaneous abortions included.

^b^ Rao-Scott χ^2^ test was used to compare proportions between HCV status; all differences were significant at *P* < .001.

^c^ Included Asian or Pacific Islander, Hispanic, Native American, and other categories as defined by the Healthcare Cost and Utilization Project; data collapsed due to small sample sizes.

Regarding hospital characteristics, the distribution of admitting hospital sizes was similar between the HCV-positive and HCV-negative cohorts. However, women who were HCV-positive more often presented in rural (14.0%; 95% CI, 12.9%-15.2% vs 11.6%; 95% CI, 10.9%-12.3%) and urban teaching (61.1%; 95% CI, 58.9%-63.4% vs 49.8%; 95% CI, 47.9%-51.6%) hospitals.

The prevalence of maternal HCV infection increased 16-fold, from 0.34 (95% CI, 0.26-0.41) cases per 1000 pregnancies in 1998 to 5.3 (95% CI, 4.9-5.7) cases per 1000 pregnancies in 2018. Over the 21-year study period, the proportion of HCV-positive pregnancies increased in all age groups: 22-fold among women aged 18 to 20 years, 31-fold among women aged 21 to 30 years, 8-fold among women aged 31 to 40 years, and 3-fold among those aged 41 to 50 years (Figure). These increases were concurrent with increasing proportions of pregnancies in women with opioid use histories (Figure; eTable 3 in Supplement 1). The group aged 21 to 30 years experienced an accelerated increase in HCV-positive pregnancies after the start of the second wave of the opioid epidemic in 2010.

**Figure.**
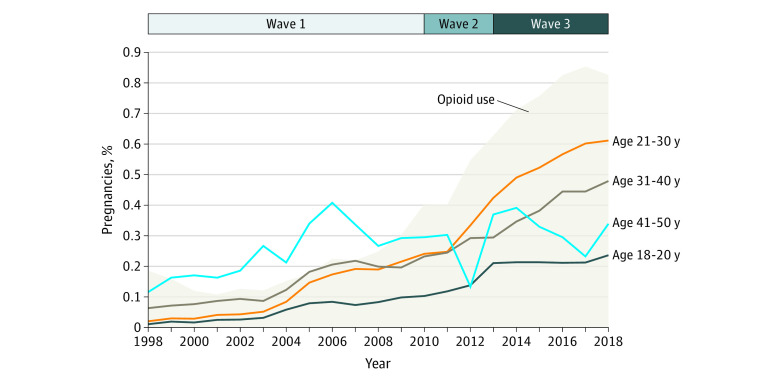
Prevalence of Hepatitis C-Positive or Opioid Use Status Among Pregnancies in the US, Across the First 3 Waves of the Opioid Epidemic

### Multivariable Analysis

After adjusting for maternal- and hospital-related covariates, maternal HCV infection was associated with higher odds of gestational hypertension (adjusted odds ratio [AOR], 1.08; 95% CI, 1.03-1.14). We found no association between maternal HCV infection and gestational anemia, gestational diabetes, preeclampsia, eclampsia, or thyroid dysfunction complicating pregnancy (Table 2).

**Table 2.** Frequencies and AORs of Maternal and Perinatal Outcomes Between HCV-Positive and HCV-Negative Groups

Variable	HCV-negative (n = 69 901 008)	HCV-positive (n = 137 259)	AOR (95% CI)^a^
Maternal outcomes			
Gestational anemia	6 525 131 (9.3)	18 046 (13.1)	0.99 (0.89-1.10)
Gestational diabetes	4 363 717 (6.2)	9049 (6.6)	1.04 (0.98-1.10)
Gestational hypertension	3 697 210 (5.3)	10 149 (7.4)	1.08 (1.03-1.14)
Preeclampsia/eclampsia	2 847 323 (4.1)	6781 (4.9)	1.00 (0.94-1.06)
Thyroid dysfunction complicating pregnancy	1 006 368 (1.4)	4564 (3.3)	1.07 (0.98-1.17)
Perinatal outcomes			
Cesarean delivery	21 133 625 (30.2)	53 047 (38.6)	1.19 (1.15-1.22)
Stillbirth	479 088 (0.7)	1339 (1.0)	0.95 (0.87-1.03)
Spontaneous abortion	1 029 547 (1.5)	2263 (1.6)	0.88 (0.82-0.95)
Preterm labor	4 755 044 (6.8)	15 892 (11.6)	1.10 (1.05-1.14)
Poor fetal growth	1 423 590 (2.0)	7858 (5.7)	1.29 (1.21-1.37)
Fetal distress	10 891 929 (15.6)	27 853 (20.3)	1.11 (1.08-1.15)
Premature rupture of membranes	3 119 340 (4.5)	8618 (6.3)	1.04 (0.99-1.10)

Abbreviations: AOR, adjusted odds ratio; HCV, hepatitis C virus.

^a^ Adjusted for maternal age (by decade), race and ethnicity, substance use (by individual categories: tobacco, alcohol, opioids, cannabis, sedatives/hypnotics, cocaine, other stimulants, hallucinogens, inhalants, other psychoactives), HIV, hemodialysis, diabetes, thyroid disorders, hypertension, anemia, Charlson-Deyo Comorbidity Index, primary payer, median household income for mother’s zip code, hospital bed size, hospital location and teaching status, and geographic region.

Concerning perinatal outcomes, maternal HCV infection was associated with higher odds of cesarean delivery (AOR 1.19; 95% CI, 1.15-1.22), preterm labor (AOR 1.10; 95% CI, 1.05-1.14), poor fetal growth (AOR 1.29; 95% CI, 1.21-1.37), and fetal distress (AOR 1.11; 95% CI, 1.08-1.15). Conversely, maternal HCV was associated with lower odds of spontaneous abortion (AOR 0.88; 95% CI, 0.82-0.95). We noted no statistically significant differences in stillbirths or premature rupture of membranes.

## Discussion

Our 21-year cross-sectional study observed a significant increase in the prevalence of HCV-positive pregnancies since the start of the US opioid epidemic in the late 1990s. Between 1998 and 2018, the greatest relative increase occurred in women aged 21 to 30 years, followed by those aged 18 to 20 years. The prevalence of HCV-positive pregnancies increased steeply in women aged 21 to 30 years during the second wave of the opioid epidemic between 2010 and 2013 (Figure), when heroin overdose–related deaths first became dominant, and continued through the third wave.^4^ Our study findings note the increase of a health care epidemic affecting women in their reproductive years in the shadow of the US opioid crisis. Overall, our data are in keeping with those of the Centers for Disease Control and Prevention, which have shown increasing cases of HCV since 2010.^3^

In our nationally representative cohort, the overall prevalence of HCV-positive pregnancies in 2018 was 5.3 cases per 1000 pregnancies. The estimate was similar to that of a recent study using the US Standard Certificate of Live Birth from 2016 to 2020.^21^ However, the earliest recommendation for universal HCV screening in pregnancy did not arrive until 2018, so our findings may have underestimated the true prevalence of HCV-positive pregnancies in the US during the study period.^9,22^ Our data source also could not differentiate between active HCV viremia vs mere HCV seropositivity, which remains long after the viremia resolves. Given an approximate 38% spontaneous viral clearance among women acutely exposed to HCV and 3.7 million births in the US in 2021,^23,24^ we conservatively estimate approximately 12 000 pregnancies yearly with HCV viremia. Thus, at a 6% risk of vertical transmission,^8^ approximately 725 infants are born with HCV infection in the US each year.

Hepatitis C virus infection has health associations that extend beyond the liver. In this study cohort, HCV-positive women scored higher on the baseline Charlson-Deyo Comorbidity Index than their HCV-negative peers. We also noted that maternal HCV infection was associated with higher odds of gestational hypertension, cesarean delivery, preterm labor, poor fetal growth, and fetal distress. In contrast, maternal HCV infection was associated with lower odds of spontaneous abortion, although potential explanatory mechanisms were elusive, as early pregnancy losses most often result from genetic anomalies.^25^ A notable historical consideration was the use of elective cesarean delivery aimed to circumvent mother-to-child HCV transmissions in the 1990s and early 2000s due to conflicting available data.^26,27^ The American College of Obstetricians and Gynecologists formally recommended against elective cesarean delivery for maternal HCV in 2007,^28^ so a time-dependent association was possible. Nonetheless, an analysis of nationally representative data from 2012 to 2018 noted increased odds of cesarean delivery in the HCV-positive group compared with HCV-negative controls.^12^ Overall, our study findings were consistent with many, albeit not all, prior studies on associations between HCV infection and maternal and perinatal outcomes. For instance, 2 meta-analyses of studies on maternal HCV infection, preterm birth, and poor fetal growth calculated pooled estimates that qualitatively concurred with our findings, but individual studies included in the meta-analyses did not always reach the same conclusions.^29,30^ Elucidating the mechanisms of perinatal HCV outcomes was ultimately outside the scope of our study; further physiologic-based investigations are needed.

Our study complemented other publications on HCV infection during pregnancy. While many existing studies showed epidemiologic snapshots of shorter lengths after the early 2000s,^6,11,12^ our analysis of a nationally representative data set provides a single, unified trend estimate over 2 decades to encompass the start of the US opioid epidemic. We also featured more granularity than earlier studies when representing substance use.^14^ Instead of a generic composite clinical factor (eg, drug use), our analysis specifically captured and adjusted for 10 individual categories of substance use (eg, alcohol, opioids, and cocaine), several of which may have higher magnitudes of association with adverse pregnancy-related outcomes. Thus, we expect our investigation of HCV-associated adverse maternal and perinatal outcomes to have less residual confounding from substance use disorders.

Our findings on the increasing prevalence of HCV-positive pregnancies and associated adverse outcomes support the recent recommendations for universal HCV screening during each pregnancy.^9,10^ Universal screening is undoubtedly more comprehensive than previous strategies of targeted screening.^22^ One cohort study reported that universal HCV screening during pregnancy identified 31% more expectant mothers with HCV seropositivity than a risk-based screening protocol.^31^ Yet, universal HCV screening of pregnant women also may remain cost-effective even when the disease prevalence is as low as 0.07%.^32^ Despite these advantages, universal screening is only one step in the HCV care cascade. Given the health-related implications and vertical transmission risk, connecting to appropriate specialist care is paramount for both mother and child. However, previous data report that postpartum engagement with HCV care occurs in only about a quarter of the cases.^31,33^

Our study findings suggest that multidisciplinary collaborations in caring for women with HCV may help enhance engagement with specialist care. In the cohort studied herein, alcohol, tobacco, and drug use and limited income were more common among HCV-positive women. Substance use and financial insecurity have been associated with higher barriers to health care access,^34,35^ increasing the risk of medical complications. Obstetric and primary care clinics can aim to establish a more durable and comprehensive care model through collaborations with hepatology or infectious disease, addiction medicine, social work, case management, and psychiatry services. A recently published perinatal care model from a safety-net hospital combining a linkage protocol and a multidisciplinary colocated clinic resulted in increased HCV treatment initiations (adjusted rate ratio, 3.36; 95% CI, 1.57-7.17).^36^ Further investigations into the optimal implementation of maternal-infant care linkage programs can help advance the mission of HCV eradication.

### Strengths and Limitations

A strength of our study is its large sample size, which allowed for sufficient statistical precision to analyze relatively infrequent events during pregnancy. The sample size enabled us to account for multiple potential confounders with our multivariable regression models. For instance, our model was able to condition on 10 individual substance categories. Nonetheless, the large sample size could also increase statistically significant findings with unclear implications in the clinical setting; therefore, informed interpretations and comparisons with other published studies are still necessary. Additionally, the NIS is nationally representative, attenuating regional practice pattern variations. We also queried and collated 21 years of data from the NIS within the same analysis to generate long-term trend estimates and create a summarizing visualization.

We acknowledge several limitations. First, *ICD-9-CM* and *ICD-10-CM* codes often do not explicitly capture the absence of a diagnosis (eg, HCV-negative) and thus can introduce misclassification bias. For example, some pregnant women with HCV may have been unscreened or miscoded at hospital discharge. Thus, the true prevalence of HCV-positive pregnancies in the US may be higher than reported herein, and our data may have underestimated the associations between maternal HCV and maternal and perinatal adverse events. However, a study of National Center for Health Statistics birth certificate data from 2011 to 2016 suggested that HCV screening practices improved over time,^37^ although it could also partly explain the increasing trend we observed. Second, the NIS does not collect data on HCV viral titers, restricting us from further defining HCV-positive as active HCV viremia or nonviremic HCV seropositivity. Consequently, our prevalence estimates likely contained a mix of both scenarios; we also could not analyze the differential outcomes of viral titer levels or resolved infection in perinatal events. Third, the NIS does not capture several potentially relevant background characteristics, such as prenatal care or health literacy, so some unmeasured or residual confounding could have influenced our statistical inferences. Fourth, the NIS reports only hospitalization data; it does not capture adverse outpatient events occurring earlier in pregnancy or during home births. However, out-of-hospital births account for fewer than 2% of all childbirths in the US.^38^ Fifth, the NIS underwent a sampling redesign that resulted in a one-time disruption of the 2012 data.^19^ Despite the revised trend weights used in our analysis,^39^ the sampling redesign likely still contributed to the unexpected single-year drop in the prevalence of HCV-positive pregnancies in 2012 among women aged 41 to 50 years. In comparison, the other 3 age cohorts were substantially larger and more robust against aberrant statistical processes. Sixth, the NIS does not link mother-to-infant medical records, so we could not assess the long-term outcomes of children born to HCV-positive mothers.

## Conclusions

This cross-sectional study noted the prevalence of maternal HCV infection has increased markedly since the start of the opioid epidemic in the US, reaching 5.3 cases per 1000 pregnancies in 2018. The most substantial increases during the opioid epidemic were noted in women aged 18 to 30 years. Additionally, HCV infection is associated with a heightened risk of adverse maternal and perinatal outcomes. Overall, our data support the recommendations for universal HCV screening with each pregnancy proposed by the Centers for Disease Control and Prevention and American College of Obstetricians and Gynecologists. Perinatal care and delivery may be the initial health care exposure for many women. These touchpoints represent an opportunity for health care professionals to identify HCV infection and link women and their children to appropriate specialist care.

## Notes

Supplement 1. **eTable 1.** *International Classification of Diseases* (*ICD*) Codes Used in the Analysis, Excluding Codes for the Charlson-Deyo Comorbidity Index **eTable 2.** Frequencies of Hepatitis C–Positive Pregnancies in the US by Year, From 1998 to 2018 **eTable 3.** Pregnancies in the US With and Without Opioid Use by Year, From 1998 to 2018

Supplement 2.
Data Sharing Statement
