# Supplementary material for: Trends in the Prevalence of Hepatitis C Infection During Pregnancy and Maternal-Infant Outcomes in the US, 1998 to 2018
Source: JAMA Netw Open. 2023 Jul 21;6(7):e2324770. doi: 10.1001/jamanetworkopen.2023.24770 (PMC10362466; doi:10.1001/jamanetworkopen.2023.24770)
Supplement: Supplement 1. — eTable 1. International Classification of Diseases (ICD) Codes Used in the Analysis, Excluding Codes for the Charlson-Deyo Comorbidity Index eTable 2. Frequencies of Hepatitis C–Positive Pregnancies in the US by Year, From 1998 to 2018 eTable 3. Pregnancies in the US With and Without Opioid Use by Year, From 1998 to 2018 [file jamanetwopen-e2324770-s001.pdf]

Supplementary Online Content

Chen PH, Johnson L, Limketkai BN, et al. Trends in the prevalence of hepatitis C infection during pregnancy and maternal-infant outcomes in the US, 1998 to 2018. *JAMA Netw Open*. 2023;6(7):e2324770. doi:10.1001/jamanetworkopen.2023.24770

eTable 1. *International Classification of Diseases* (*ICD*) Codes Used in the Analysis, Excluding Codes for the Charlson-Deyo Comorbidity Index

eTable 2. Frequencies of Hepatitis C–Positive Pregnancies in the US by Year, From 1998 to 2018

eTable 3. Pregnancies in the US With and Without Opioid Use by Year, From 1998 to 2018

This supplementary material has been provided by the authors to give readers additional information about their work.

© 2023 Chen PH et al. *JAMA Network Open.*

eTable 1. International Classification of Diseases (ICD) codes used in the analysis, excluding codes for the Charlson-Deyo Comorbidity Index

<table>
<tr><th colspan="2">Diagnosis</th><th>ICD-9-CM codes</th><th>ICD-10-CM/PCS codes</th></tr>
<tr><td colspan="2">Childbirth (vaginal and Cesarean)</td><td>Diagnosis codes: 641.*1, 642.*1, 642.*2, 643.*1, 644.21, 645.*1, 646.*1, 646.*2, 647.*1, 647.*2, 648.*1, 648.*2, 649.*1, 649.*2, 650, 651.*1, 652.*1, 653.*1, 654.*1, 654.*2, 655.*1, 656.*1, 657.01, 658.*1, 659.*1, 660.*1, 661.*1, 662.*1, 663.*1, 664.*1, 665.*1, 665.22, 666.*2, 667.*2, 668.*1, 668.*2, 669.*1, 669.*2, 670.*2, 671.*1, 671.*2, 672.02, 673.*1, 673.*2, 674.*1, 674.*2, 675.*1, 675.*2, 676.*1, 676.*2, 678.*1, 679.*1, 679.*2, V27.*, V30.*-V37.*, V39.*
Procedure codes: 72.*, 73.*, 74.0-74.2, 74.4, 74.9*, 75.4</td><td>CM: O10.*2, O11.4, O12.*4, O13.4, O14.*4, O16.4, O24.*2, O25.2, O26.62, O26.72, O42.*2, O60.22*, O60.23*, O63.2, O66.5, O67.*, O68, O69.*, O70.0-O70.3, O70.9, O74.*, O75.0, O75.1, O75.5, O75.8*, O75.9, O76, O77.*, O80, O82.*, O88.*2, O98.*2, O99.12, O99.214, O99.284, O99.3*4, O99.42, O99.52, O99.62, O99.72, O99.8*4, O9A.*2, Z37.0, Z37.2, Z37.3, Z37.5*, Z37.6*, Z37.9, Z38.*
PCS: 0W8N*, 1090*, 10D0*, 10D1*, 10E0*</td></tr>
<tr><td colspan="2">Viral hepatitis C</td><td>070.41, 070.44, 070.51, 070.54, 070.7*, V02.62</td><td>B17.1*, B18.2, B18.8, B19.2*, Z22.52</td></tr>
<tr><td colspan="4">Covariates:</td></tr>
<tr><td rowspan="10">Substance use</td><td>Tobacco</td><td>305.1, 649.0*</td><td>F17.*, T65.2*, O99.33*, Z72.0</td></tr>
<tr><td>Alcohol</td><td>291.*, 303.*, 305.0*, 790.3, 980.0, E860.0, E860.1, V11.3</td><td>F10.*, O99.31*, R78.0, T51.0*</td></tr>
<tr><td>Opioids</td><td>304.0*, 304.7*, 305.5*, 965.0*, E850.0-E850.2</td><td>F11.*, P04.14, R78.1, T40.0*-T40.4*, T40.6*</td></tr>
<tr><td>Cannabis</td><td>304.3*, 305.2*</td><td>F12.*, P04.81, T40.7*</td></tr>
<tr><td>Sedatives, hypnotics, or anxiolytics</td><td>304.1*, 305.4*, 967.*, 969.1-969.5, E851.*-E853.*, E950.1-E950.3, E980.1-E980.3</td><td>F13.*, T42.3*-T42.4*, T42.6*-T42.7*, P04.17, P04.1A</td></tr>
<tr><td>Cocaine</td><td>304.2*, 305.6*, 760.75, 970.81</td><td>F14.*, R78.2, T40.5*, P04.41</td></tr>
<tr><td>Other stimulants</td><td>304.4*, 305.7*, 969.7*, E854.2</td><td>F15.*, T43.6*, P04.16</td></tr>
<tr><td>Hallucinogens</td><td>304.5*, 305.3*, 969.6, E854.1</td><td>F16.*, P04.42, R78.3, T40.8*-T40.9*</td></tr>
<tr><td>Inhalants</td><td>Not available</td><td>F18.*</td></tr>
<tr><td>Other psychoactives</td><td>304.8*, 305.8*, E854.8, 969.0*, 969.8, 969.9</td><td>F19.*, R78.5, T43.8*, T43.9*</td></tr>
<tr><td colspan="2">HIV</td><td>042, 043, 044</td><td>B20, B21, B22, B23, B24</td></tr>
<tr><td colspan="2">On hemodialysis</td><td>585.6, V45.11</td><td>N18.6, Z99.2</td></tr>
</table>

© 2023 Chen PH et al. *JAMA Network Open.*

Diabetes mellitus	250.0*-250.7*	E10.1*-E10.5*, E10.9, E11.1*-E11.5*, E11.9, E13.1*-E13.5*, E13.9, E14.1*-E14.5*, E14.9
Thyroid disorders	240.*-246.*	E00.*-E07.*
Hypertension	401.*-405.*	I10.*, I15.*, I16.*
Anemia	280.*-285.*	D50.*-D64.*
Maternal outcomes:		
Anemia complicating pregnancy	648.2*	O99.0*
Gestational diabetes	648.0*, 648.8*	O24.*
Hypertension complicating pregnancy, minus preeclampsia	642.0*-642.3*, 642.9*	O10.*, O13.*, O16.*
Preeclampsia/eclampsia	642.4*-642.7*	O11.*, O14.*, O15.*
Thyroid dysfunction complicating pregnancy	648.1*	O99.28*
Perinatal outcomes:		
Cesarean delivery	Diagnosis codes: 669.7*<br>Procedure codes: 74.0-74.2, 74.4, 74.99	CM: O82.*, O84.2<br>PCS: 10D00Z*
Stillbirth	656.4*, V27.1, V27.4, V27.7	O36.4X*, Z37.1, Z37.4, Z37.7
Spontaneous abortion	631.0, 631.8, 632, 634.*, 637.*, 638.*, V27.1, V27.3, V27.4, V27.7	O02.*, O03.1, O03.30-O03.36, O03.38, O03.39, O03.4, O03.6, O03.7, O03.80-O03.86, O03.88, O03.89, O03.9
Preterm labor	644.*	O60.*
Long labor	662.*	O63.*
Poor fetal growth	656.5*	O36.5*
Fetal distress	656.3*, 656.8*, 659.7*	O68.*, O76.*, O77.*
PROM	658.1*	O42.*

Abbreviations: CM, Clinical Modification; PCS, Procedure Coding System; HIV, human immunodeficiency virus; PROM, premature rupture of membranes

© 2023 Chen PH et al. *JAMA Network Open.*

eTable 2. Frequencies of hepatitis C-positive pregnancies* in the US by year, from 1998 to 2018

	Deliveries	Stillbirths	Spontaneous Abortions	Caesarean sections
**1998**	1206	6	22	313
**1999**	1584	16	41	495
**2000**	1794	0	24	569
**2001**	2168	14	35	759
**2002**	2377	30	65	843
**2003**	2530	31	62	974
**2004**	3946	44	76	1428
**2005**	6226	63	166	2476
**2006**	7265	56	176	3001
**2007**	8262	101	153	3396
**2008**	7476	60	97	2853
**2009**	7913	50	95	3300
**2010**	8389	94	176	3403
**2011**	8692	94	195	3450
**2012**	2760	45	65	1085
**2013**	3985	55	90	1435
**2014**	5965	70	115	2215
**2015**	8190	80	155	3165
**2016**	11535	80	95	4605
**2017**	14775	140	160	5915
**2018**	19085	210	200	7365
**Total**	136121	1339	2263	53047

*Due to overlapping codes, the categories of deliveries, stillbirths, spontaneous abortions, and Caesarean sections are not mutually exclusive.

© 2023 Chen PH et al. *JAMA Network Open.*

eTable 3. Pregnancies* in the US with and without opioid use by year, from 1998 to 2018

	Opioid use	No opioid use	%
**1998**	6738	3625028	0.185529574
**1999**	5929	3726197	0.158863875
**2000**	4786	3967743	0.120477409
**2001**	4201	3883489	0.108059027
**2002**	5116	4032284	0.126715213
**2003**	4811	3955628	0.121476432
**2004**	6279	4117468	0.152264433
**2005**	7172	4090242	0.175037231
**2006**	9376	4154669	0.225165674
**2007**	9726	4405200	0.220298143
**2008**	10232	4106097	0.248570996
**2009**	12182	4011639	0.302747066
**2010**	15293	3774610	0.403519562
**2011**	15118	3743456	0.402227015
**2012**	5165	938805	0.547157219
**2013**	7075	1121360	0.626974527
**2014**	10390	1445980	0.713417607
**2015**	14130	1853945	0.756393614
**2016**	19485	2342868	0.824813226
**2017**	25015	2906857	0.853209144
**2018**	30010	3600239	0.826665058

*Includes deliveries and spontaneous abortions

© 2023 Chen PH et al. *JAMA Network Open.*
