# Supplementary material for: Trends in the Prevalence of Hepatitis C Infection During Pregnancy and Maternal-Infant Outcomes in the US, 1998 to 2018
Source: JAMA Netw Open. 2023 Jul 21;6(7):e2324770. doi: 10.1001/jamanetworkopen.2023.24770 (PMC10362466; doi:10.1001/jamanetworkopen.2023.24770)
Supplement: Supplement 2. — Data Sharing Statement [file jamanetwopen-e2324770-s002.pdf]

Data Sharing Statement

Chen. Trends in the Prevalence of Hepatitis C Infection During Pregnancy and Maternal-Infant Outcomes in the US, 1998 to 2018. *JAMA Netw Open*. Published July 21, 2023. doi:10.1001/jamanetworkopen.2023.24770

Data
Data available: No

Additional Information
Explanation for why data not available: Healthcare Cost and Utilization Project data are publicly available from the Agency for Healthcare Research and Quality at https://www.hcup-us.ahrq.gov.
